# Rapid Fabrication of Graphene Field-Effect Transistors with Liquid-metal Interconnects and Electrolytic Gate Dielectric Made of Honey

**DOI:** 10.1038/s41598-017-10043-4

**Published:** 2017-08-31

**Authors:** Richard C. Ordonez, Cody K. Hayashi, Carlos M. Torres, Jordan L. Melcher, Nackieb Kamin, Godwin Severa, David Garmire

**Affiliations:** 1 University of Hawai’i at Mānoa, Department of Electrical Engineering, Honolulu, HI 96822 USA; 2 Space and Naval Warfare Systems Center Pacific, Pearl City, HI 96782 USA; 3 Space and Naval Warfare Systems Center Pacific, San Diego, CA 92152 USA; 4 Hawai’i Natural Energy Institute, Honolulu, HI 96822 USA

## Abstract

Historically, graphene-based transistor fabrication has been time-consuming due to the high demand for carefully controlled Raman spectroscopy, physical vapor deposition, and lift-off processes. For the first time in a three-terminal graphene field-effect transistor embodiment, we introduce a rapid fabrication technique that implements non-toxic eutectic liquid-metal Galinstan interconnects and an electrolytic gate dielectric comprised of honey. The goal is to minimize cost and turnaround time between fabrication runs; thereby, allowing researchers to focus on the characterization of graphene phenomena that drives innovation rather than a lengthy device fabrication process that hinders it. We demonstrate characteristic Dirac peaks for a single-gate graphene field-effect transistor embodiment that exhibits hole and electron mobilities of 213 ± 15 and 166 ± 5 *cm* ^2^/*V*·*s* respectively. We discuss how our methods can be used for the rapid determination of graphene quality and can complement Raman Spectroscopy techniques. Lastly, we explore a PN junction embodiment which further validates that our fabrication techniques can rapidly adapt to alternative device architectures and greatly broaden the research applicability.

## Introduction

The lowering cost of graphene synthesis has created opportunities that include lightweight electronics such as wearable, flexible, and electromagnetic sensors^1^. However, to explore such scientific phenomena one must be trained and certified in sophisticated optical characterization and microfabrication techniques to design and fabricate graphene devices such as graphene field-effect transistors (GFETs). Furthermore, the complexity of the required fabrication steps must be conducted in a controlled environment to increase device yield and minimize exposure to harmful chemicals. This process can be quite time-consuming and demanding depending on the scope of the device architecture and the allotted time given for expenditure of project funds. Such drawbacks reduce the accessibility of graphene research across academic and private institutions.

In a typical GFET fabrication process, graphene is synthesized via chemical vapor deposition and transferred onto a target substrate via delamination and the graphene quality is measured via Raman Spectroscopy. Lift-off processes are then used to construct contact electrodes, gate-dielectrics, and gate-contacts^2–7^. Unfortunately, numerous articles have reported degradation in graphene transistor performance due to mechanical and electrical strain put on graphene that is a direct result of photolithography and physical vapor deposition^8–10^. In addition, graphene has been shown to exhibit high contact resistance with standard electrode materials relative to its size that create non-ideal performance across the board^11^. This performance limitation will potentially force a researcher back into the cleanroom to identify drawbacks in his or her fabrication process.

In this paper, we demonstrate the rapid fabrication of a three-terminal graphene-field effect transistor embodiment with the use of commercially available eutectic liquid-metal Galinstan interconnects and electrolytic gate dielectric comprised of honey (*LM-GFET*). We demonstrate comparable GFET performance with the proposed inexpensive, non-traditional materials to much more common ionic gel materials^12, 13^. Galinstan is a non-toxic liquid-metal alloy consisting of 68.5% gallium, 21.5% indium, and 10% tin^14^ that possesses desirable conformal properties. In a previous study, Galinstan device interconnects exhibited less than a 5.5% change in resistance for a graphene two-terminal device when subjected to repeated deformations as small as 4.5 mm radius of curvature^15^. Moreover, graphene transistors can greatly benefit from the conductivity of Galinstan (2.30 × 10^6^ *S*/*m*), a desirable vapor pressure (<1 × 10^−6^ *Pa* at 500 °*C*) compared with mercury (0.1713 *Pa* at 20 °*C*), and a stable liquid state across a broad temperature range (−19 °*C* to 1300 °*C*)^16^. Honey is typically produced via sugary secretions from bees, harvested, and packaged under various brand names for commercial food consumption. Honey contains various concentrations of water, vitamins, minerals, amino acids, and sugars: fructose, glucose, sucrose that can be controlled via bee production and honey extraction techniques^17^. To our benefit, honey formulates an ionic gel-like solution analogous to ion gels. Ion gels consist of room-temperature ionic liquids and gelating triblock copolymers^12, 13^. Recently, ion gels have demonstrated ideal performance as electrolytic gate dielectrics for flexible GFET devices due to an ability to produce extremely high capacitance and high dielectric constants required for high on-current and low-voltage operation^12^. The introduction of honey as an electrolytic gate dielectric is advantageous for rapid fabrication of GFET devices due to the commercial availability, non-toxicity, control of ionic content that can be used to alter dielectric properties, and quick mixing that reduces preparation time of honey. Currently, ion gels require special preparation in an atmospheric controlled environment to mitigate outgassing and combustion, which only adds to its complexity.

## Results and Discussion

### Transistor Architecture and Operation

**Figure 1:**
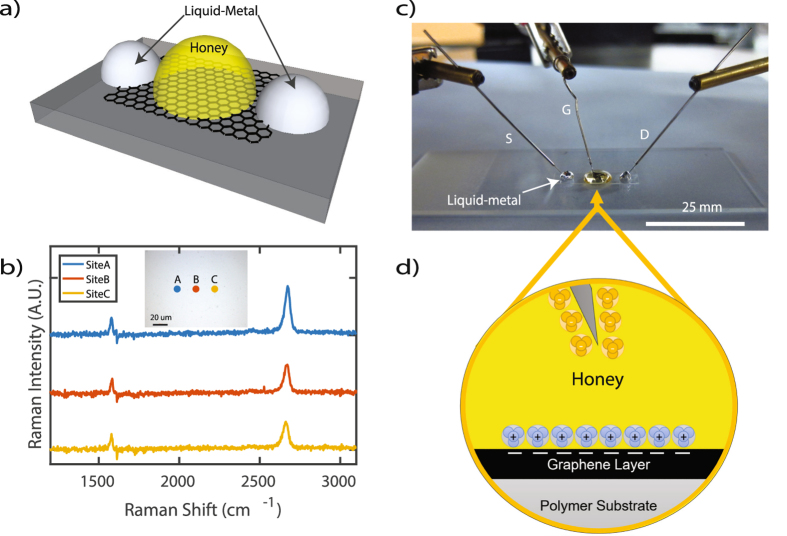
(**a**) Illustration of a graphene field-effect transistor with liquid-metal source and drain electrodes (*LM-GFET*); and honey. (**b**) Raman spectroscopy profile of graphene sample transferred to Polyethylene Terephthalate. Inset: Location of sites A–C. (**c)** Image of the *LM-GFET* and (**d**) representation of charge distribution in electrolytic gate dielectrics comprised of honey.

The transistor architecture of the *LM-GFET* device is shown in Fig. 1a and an image of the *LM-GFET* is shown in Fig. 1c. The device is comprised of liquid-metal Galinstan source and drain electrodes that are overlaid on monolayer graphene transferred to Polyethylene Terephthalate (PET). A detailed fabrication process for the *LM-GFET* device is described in the Methods Section. Honey, was used as a gel-like electrolytic gate dielectric to generate an enhanced electric field-effect response above graphene. Due to the presence of ions and high polarizability of honey, a diffusion of charge is formed at the thin layer between honey and graphene. This layer forms an electric double layer and is typical when ionic liquids contact conductive materials^12, 13^. Due to the nanoscale separation distance of the electric double layer, usually from 1-10 *nm*, a large charge gradient is formed on the surface of graphene. For example, in the case a gate electrode is positively charged and submerged in honey, anions will accumulate at the gate/honey interface and cations will accumulate at the honey/graphene interface. The resultant electrical double layer at the honey/graphene interface will then alter the graphene conductivity, Fig. 1d. The opposite is true for the case in which the gate electrode is negatively charged.

### Single-Gate Transistor Characteristics

**Figure 2:**
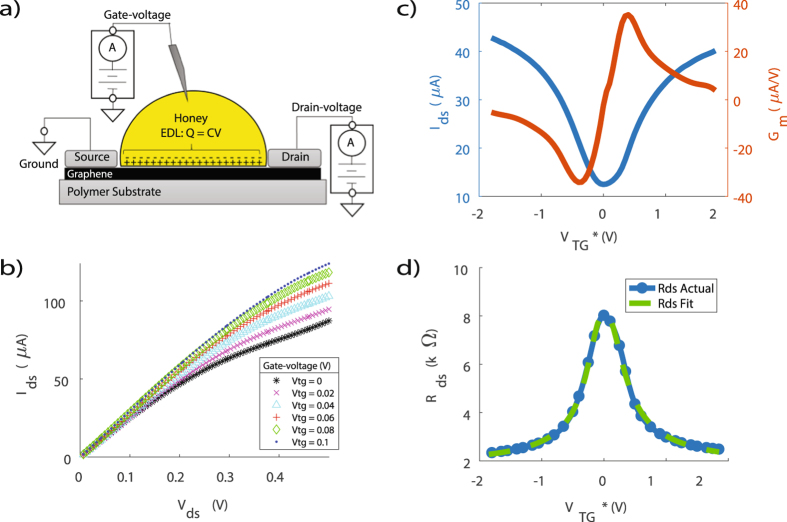
(**a**) Schematic representation of a *LM-GFET* device with a single-gate and location of the electric double layer. (**b**) Drain-to-source current as a function of drain-to-source voltage for varied top-gate voltage (*I*_*ds*_ − *V*_*ds*_). (**c**) *Left*: Relationship of drain-to-source current as a function of top-gate voltage $({I}_{ds}-{V}_{TG}^{\ast })$ and *Right* : Transconductance, *G*_*m*_, as a function of top-gate voltage $({G}_{m}-{V}_{TG}^{\ast })$. (**d**) Model fit overlaid on drain-to-source resistance as a function of top-gate voltage $({R}_{ds}-{V}_{TG}^{\ast })$.

A schematic representation of the electrical measurements is shown in Fig. 2a. Fig. 2b–d illustrates graphene transport characteristics for a single-gated *LM-GFET* device. The V-shaped curve of the relationship between top-gate voltage ${V}_{TG}^{\ast }$ and drain-to-source current *I*_*ds*_ in Fig. 2c highlights the ambipolar operation that is characteristic of any graphene field-effect transistor, and provides designers the flexibility to bias the device in either hole or electron conduction mode. A well-documented model extraction technique was utilized to extract graphene parameters from the device’s transfer curve^18^. The model fit in Fig. 2d determined hole and electron mobilities of 213 ± 15 and 166 ± 5 *cm* ^2^/*V* · *s* respectively, at a drain bias of 100 *mV*. Despite the rapid and inexpensive fabrication process, the *LM-GFET* devices exhibited performance comparable to that of much more elaborately fabricated GFET device^1, 12, 13^. In addition, Fig. 2c illustrates the device’s transconductance that reaches a considerable value of 38 *μA*/*V* with a large degree of symmetry and linearity within the operational range of −0.5 *V* to 0.5 *V*. This linear, ambipolar transconductance has significant utility in ambipolar electronic circuits such as radio frequency mixers, digital modulators, and phase detectors^19^. Figure 2b illustrates the *I*_*ds*_ − *V*_*ds*_ response to the various transconductances associated with different ${V}_{TG}^{\ast }$ values. Fortunately, due to the monotonic transconductance of the *LM-GFET* devices, the drain-to-source voltage *V*_*ds*_ sweep does not demonstrate an inflection point. Particularly, the varying ${V}_{TG}^{\ast }$ curves do not intersect one another, which cannot be presumed to occur in all standard GFET devices^20^. Instead, the *I*_*ds*_ − *V*_*ds*_ trend is encouraging because the drain currents diverge at higher *V*_*ds*_ biases. In a GFET sensor application, a designer can first bias their device at a desired *V*_*ds*_ and *I*_*ds*_. Therefore, an external stimuli can trigger a change in the device’s gate voltage, which will create a significant change in *I*_*ds*_. Note, the dielectric constant of honey was measured to be 21 and the gate capacitance was measured to be 2.3 *μF*/*cm* ^2^ using a LCR meter which is very comparable to what is described in literature^21, 22^.

### Comparison of Ion Gel with an Electrolytic Gate Dielectric Made of Honey

**Figure 3:**
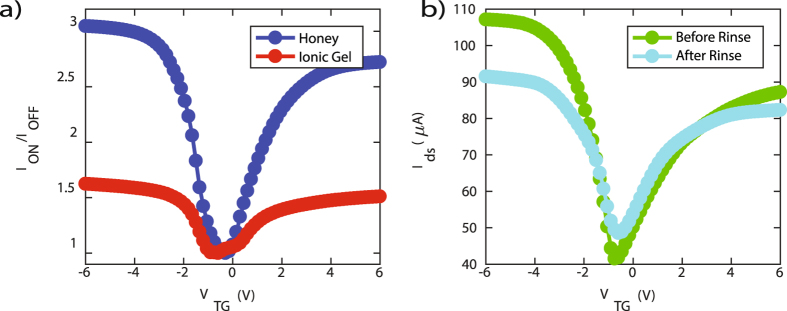
(**a**) Comparison of drain-to-source current, *ON*/*OFF*, ratio as a function of top-gate voltage (*I*_*ON*_/*I*_*OFF*_ − *V*_*TG*_) for a *LM-GFET* with an electrolytic gate dielectric comprised of honey (blue) and ion gel (red), *V*_*ds*_ = 1.2*V*. (**b**) Comparison of drain-to-source current as a function of top-gate voltage (*I*_*ds*_ − *V*_*TG*_) for a *LM-GFET* before (green) and after (cyan) rinsing of the honey and liquid metal electrodes.

Transport characteristics were compared with an ion gel *LM-GFET* to validate the use of honey as a electrolytic gate dielectric. The ion gel used was comprised of 1-Ethyl-3 Methylimidizalium Bis(Trifluoromethylsufonyl)imide ([EMIM][TFSI]) ionic liquid in Polystyrene-Poly (Ethylene Oxide) (PS-PEO) diblock copolymer. A detailed description of the ion gel synthesis is given in the Methods section. Figure 3a, illustrates the difference in the drain-to-source current, *ON/OFF*, ratio as a function of top-gate voltage (*I*_*ON*_/*I*_*OFF*_ − *V*_*TG*_). The transport characteristics are presented as a ratio of *I*_*ON*_ and *I*_*OFF*_ due to the significant difference in drain-to-source current between the sample measurements. The maximum operating current of the device with ion gel was 124 ± 10 *μA* with an *I*_*ON*_/*I*_*OFF*_ = 1.7, where *V*_*ds*_ = 1.2 *V*. The maximum operating current of the device with honey was 620 ± 40 *μA* with an *I*_*ON*_/*I*_*OFF*_ = 3.1. A model fit determined the hole and electron mobilities for the ion gel device was 61 ± 3 and 189 ± 3 *cm* ^2^/*V* · *s* and 100 ± 4 and 126 ± 5 *cm* ^2^/*V* · *s* respectively for the Honey *LM-GFET*.

The importance of a high capacitance electrolytic gate dielectric became clear in our comparison of the honey and ion gel *ON/OFF* ratios. The high capacitance of honey (~2 *μF*/*cm* ^2^) enabled a significant electric field-effect, hence enabled a large drain current. The opposite was seen for the lower capacitance of the ion gel (0.1 *μF*/*cm* ^2^). The reduced field-effect of the ion gel device may be limited by a relatively high gate leakage current that is typical of electrolytic gate dielectrics^23^. The gate leakage current of the ion gel was measured to be 20 *μA*, which was 4*x* greater than the honey. Although, the gate leakage was high for the ion gel device, the formation of the electric double layer at the graphene/ion gel interface allowed for ambipolar operation. In addition, the dirac peaks measured for ion gel devices were not as sharp as the dirac peaks measured by the honey devices. This phenomenon may be indicative of charge inhomogeneity at the electric double layer surface and can be caused by improper/incomplete mixing of the ionic liquid and copolymer solutions.

Due to the liquid properties of honey and liquid-metal, these materials may be rinsed in boiling deionized water after initial measurements have been completed. The authors include an investigation of the electrical performance of graphene before and after rinsing of the liquid-metal electrodes and electrolytic gate dielectric made of honey. It was determined there is a slight change in the electrical performance of the graphene devices after rinsing, Fig. 3b. Before rinsing the extracted hole and electron branch resistances was 917 ± 6 Ω and 1062 ± 1 Ω. After rinsing the extracted electron and hole branch resistances increased to 1065 ± 15 Ω and 1170 ± 20 Ω. The slight increase in resistance is assumed to be due to trace amounts of liquid metal residue that remained after rinsing. The liquid metal residue gradually oxidizes over time and contributes to a parasitic resistance at the contacts. Future efforts can investigate dual-rinse processes that include weak solvents followed by a DI water rinse.

Despite a DI water rinse, there is no significant shift of the charge neutrality point (Dirac peak). Additionally, the mobilities extracted from the model fit show negligible, if any, degradation. The hole and electron mobilities before rinsing the LM-GFET are 46 ± 6 and 189 ± 1 *cm* ^2^/*V* · *s* respectively at a drain bias of 100 *mV*. After rinsing, hole and electron mobilities are 88 ± 3 and 165 ± 5 *cm* ^2^/*V* · *s* respectively, at a drain bias of 100 *mV*. Notably, the extracted hole mobility increased, while the electron mobility slightly decreased. While this can be due to the nominal variance of each of the extracted values, it is believed that the DI water with subsequent heating removed several charge impurities that would have otherwise contributed to cross-sections of electron/hole scattering^24^. Therefore, the experimental results suggest that the proposed rinse process minimally impacts device performance, and yet allows designers to rapidly and prudently explore new device architectures with the same graphene material. The reuse of graphene is an incentive to reduce carbon waste.

### Rapid Characterization of Graphene Quality to Aid Raman Spectroscopy

Raman spectroscopy is the industry standard for graphene characterization and provides a researcher with the number of graphene layers, as well as the impurities or dopants present within a graphene material^25^. However, the equipment required to perform these measurements is costly, and the measurements can be time-consuming. Due to the optical magnification necessary for spatially dependent graphene Raman measurements, investigations are limited to a few grain boundaries. Moreover, inhomogeneity across graphene due to topographical imperfections and varying concentration of dopants can change quite drastically in large scale devices^26^. In GFET devices, charge carriers encounter numerous grain boundaries from source to drain. Transport characteristics such as Current-Voltage (I–V) measurements average many grain boundaries and enable a way to analyze the electrical performance of large graphene channels (on the order of several hundreds of microns).

**Figure 4:**
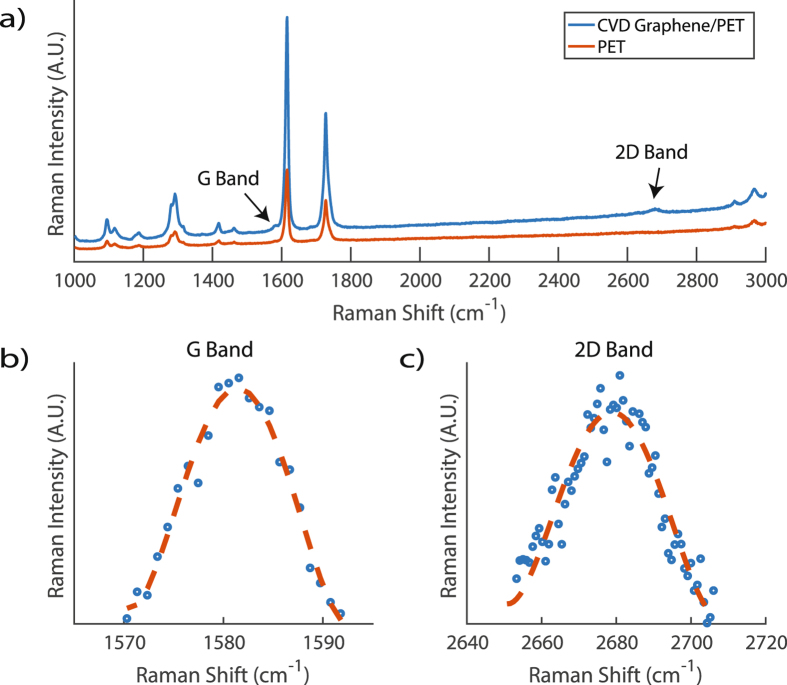
(**a**) Raman spectroscopy profile of a graphene sample transferred to Polyethylene Terephthalate (PET) (blue) and PET substrate (red). The location of the *G* Band and 2*D* Band are labeled with arrows. (**b**) Static measurement of graphene *G* Band with curve fit. (**c**) Static measurement of graphene 2*D* Band with curve fit.

Raman spectroscopy of graphene transferred onto polymer substrates is quite challenging for unspecialized laboratories. The reason being, there exist strong polymeric vibrational modes thousands of times more sensitive to Raman scattering near the *G* band of graphene, Fig. 4a. Moreover, the *G* Band is not easily identifiable and one must take great care when analyzing Raman data. To identify the *G* band, static measurements are required and consist of several prolonged exposure acquisitions that increase the signal-to-noise ratio^27^, Fig. 4b. Furthermore, subtraction techniques are required to remove the background PET Raman signature, so that, the graphene (*I*_2*D*_/*I*_*G*_) ratios can be computed to extract the number of graphene layers. The authors’ best attempt for Raman Spectroscopy characterization of graphene on PET with post-processing to remove the PET signature took approximately 1 hour per sample for a single spot. To conduct Raman measurements of three separate samples (which is the industry standard) will take up to almost 3 hours with post-processing included. Moreover, a Raman Spectroscopy map of a graphene channel will take much longer.

The authors utilized their proposed *LM-GFET* rapid fabrication methods to compare graphene on PET samples with both high and low quality that were previously determined via 514.5 *nm* Raman spectroscopy. The intention for this experiment was to validate the use of our methods as a useful tool to complement Raman Spectroscopy data for large scale graphene devices. As previously discussed, Fig. 1b illustrates Raman spectroscopy measurements for the graphene sample used in the single-gate transfer characteristics section and illustrated in Fig. 2. The extracted (*I*_2*D*_/*I*_*G*_) ratios for Sites A–C are 3.10, 1.92, and 2.13 respectively, thus indicated high quality monolayer graphene. As was determined from I–V measurements, the hole and electron mobilities for the high quality graphene was on the order of 213 ± 15 and 166 ± 5 *cm* ^2^/*V* · *s* respectively for a drain bias of 100 *mV*. On the other hand, the extracted (*I*_2*D*_/*I*_*G*_) ratios for a sample of low quality graphene was determined to be 2.44, 1.52, and 0.70 respectively. Despite being labeled low quality via the Raman spectroscopy measurements, our rapid fabrication methods determined the hole and electron mobilities were 128 ± 4 and 101 ± 4 *cm* ^2^/*V* · *s*. Although, the computed mobilities are lower than what was measured in the high quality samples, the low quality samples were respectively still quite comparable.

It has become common practice by commercial graphene manufacturers to provide graphene quality via Raman spectroscopy data of a few (1–3) spots with purchased graphene samples. Due to comparable results for the high and low quality graphene samples, there is reason to believe that our methods can complement Raman Spectroscopy measurements of large samples. One may consider graphene to be of low quality immediately after undesirable Raman spectroscopy measurements, and therefore may not utilize and dispose of the graphene sample. Our proposed methods provide an additional metric to explore graphene quality beyond spatially dependent Raman spectroscopy and in a transistor embodiment. The devices in this paper were relatively large. Users simply need to perform any I–V measurements of their fabricated devices, then use an automated code to extract electrical performance. The extracted mobility can be correlated to graphene quality as previously demonstrated^25^, and the location of the Dirac peak can provide crucial information on the impurities present.

### PN Junction Transistor Architecture and Operation

FETs operate by the formation of junctions through an inherent electric field within a bulk semiconductor. The electric field can be created via chemical doping, yet will often be fixed upon fabrication. One can create a similar adaptive effect in a GFET device by manufacturing a lateral electric field with collinear wires suspended above graphene either in a dielectric, electrolyte, or slightly conductive liquid. Moreover, the electric field does not need to be referenced to graphene or any gate electrodes.

**Figure 5:**
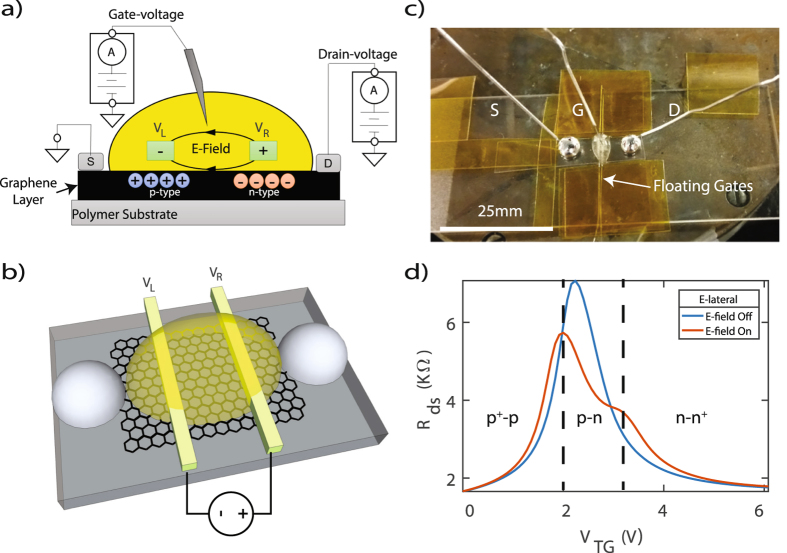
(**a**) Schematic representation of a graphene PN junction driven by an embedded lateral electric field in honey. (**b**) Visualization of PN Junction LM-GFET embodiment. (**c**) Location of liquid-metal Galinstan droplets, honey, and collinear E-field wire generators above graphene. (**d**) Graphene PN Junction characteristics as illustrated by the relationship of the drain-to-source resistance as a function of top-gate voltage (*R*_*ds*_ − *V*_*TG*_) for cases when a lateral electric field (E-lateral) is turned on and off. The sudden changes in *R*_*ds*_ illustrate changes in carrier concentration throughout the graphene channel.

An assessment of such a transistor comprised of graphene and honey, shows that the newly formed lateral electric field (E-lateral) creates an effective PN junction because any free electrons within the graphene channel are swept to one side of the channel and any holes are swept to the other end. A representation of this idea is illustrated in Fig. 5a and b and an image of the device is shown in Fig. 5c. In the event the graphene PN junction were forward biased, a higher concentration of holes will be attracted to the negative terminal and a higher concentration of electrons will be attracted to the positive terminal. Figure 5d, illustrates the fundamental graphene PN junction transport properties for when the electric field generator is switched from off to on. For the case when the E-field remains off, a relationship of *R*_*ds*_ − *V*_*TG*_ demonstrates typical GFET transport characteristics with a single Dirac peak. However, as the E-lateral is turned on, two Dirac peaks occur and the charge carrier distribution can be clearly identified.

### PN Junction Transistor Characteristics

**Figure 6:**
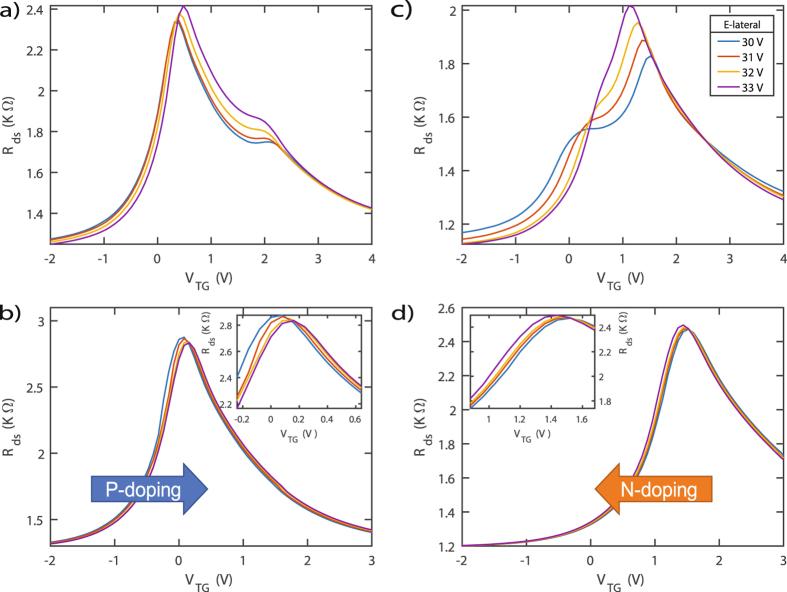
Drain-to-source resistance as a function of top-gate voltage (*R*_*ds*_ − *V*_*TG*_) for (**a**) forward bias [−*V*_*L*_, +*V*_*R*_], (**b**) both negative [−*V*_*L*_, −*V*_*R*_], (**c**) reverse bias [+*V*_*L*_, −*V*_*R*_], and (**d**) both positive [+*V*_*L*_, +*V*_*R*_] when the source of Fig. 5a is set to ground. (**a**,**c**) Change in the amplitude and separation of Dirac peaks as the lateral electric field potential (E-lateral) was varied from 30–33 *V*. (**b**) Illustration of P-type doping as *V*_*L*_ and *V*_*R*_ are set to negative voltages. (**d**) Illustration of n-type doping as *V*_*L*_ and *V*_*R*_ are set to positive voltages. Insets: Magnified view of the Dirac peak shifts.

We further demonstrate PN junction phenomena of four separate cases. Case A (Fig. 6a): Left terminal *V*_*L*_ of Fig. 5a is set to a negative voltage and the right terminal *V*_*R*_ is set to a positive voltage. This creates a forward bias scenario when the source is set to ground. For example, when *V*_*L*_ = −15 *V* and *V*_*R*_ = +15 *V*, E-lateral = 30 *V* between the two terminals. Case B (Fig. 6b): *V*_*L*_ and *V*_*R*_ are set to negative voltages. Case C (Fig. 6c): *V*_*L*_ is set to a positive voltage and *V*_*R*_ is set to a negative voltage, therefore creating a reverse bias scenario. Finally, Case D (Fig. 6d): *V*_*L*_ and *V*_*R*_ are set to positive voltages.

As E-lateral was turned on and *V*_*TG*_ was varied in Case A and Case C, dual Dirac peaks indicated the presence of two charge regions along the graphene channel. The amplitude and distance between the two Dirac peaks were than controlled by altering E-lateral from 30–33 *V*. This effect can be attributed to charge inhomogeneity within the graphene landscape that was driven by E-lateral. Such techniques are applicable in photodetector applications as one could carefully control optical transitions with an applied electric field. As the lateral electric field strength is altered, the distance between the two Dirac peaks changes. Moreover, the distance between the Dirac peaks indicates the work function required to generate photocurrent which is governed by the Fermi energy level ${E}_{F}=\hslash {v}_{F}\sqrt{\pi n}$ and whether optical interband transitions are allowed^28, 29^. More noticeably in Case C, as E-lateral was increased, the dual Dirac Peaks merge into a single Dirac Peak. This effect, worthy of future study, may be attributed to non-linear screening of the spatial charge inhomogeneity within the graphene channel. Perhaps for Case C the charge inhomogeneity is of lower density, therefore, screening is more effective with E-lateral.

There was a noticeable shift in the overall transport characteristics of Case A and Case C. To explore this phenomena, Case B and Case D demonstrate cases in which *V*_*L*_ and *V*_*R*_ are biased both negative and both positive, respectively. With respect to the intrinsic doping concentration, it was demonstrated that graphene underwent slight p-type doping (right shift) as E-lateral was biased more negatively. This effect can be attributed to an excess of charge carrier diffusion to the electric double layer that increases with E-lateral^30^ and was similarly seen for a single-gated *LM-GFET* device operation. The opposite is true when the *V*_*L*_ and *V*_*R*_ were biased both positive and n-type doping occurred (right shift). An additional effect of biasing is the change in the drain-to-source resistance *R*_*ds*_ maximum as the lateral electric field strength is altered, Inset of Fig. 6b and d.

### Summary

Our transistors have demonstrated a method to rapidly characterize graphene materials with the use of non-toxic eutectic liquid-metal Galinstan interconnects and honey gate dielectrics in a three-terminal graphene field-effect transistor embodiment. The devices characterized in the paper were fabricated within less than 30 minutes and in a general laboratory setting. Our methods are repeatable, therefore one can adopt our methods into an automated quality assurance process at a per chip level for end-to-end fabrication increasing yield and eliminating tedious testing. Despite not being fabricated in a conventional cleanroom, our devices provided comparable performance to the current state-of-the-art. We demonstrated transport characteristics for a single-gate graphene field-effect transistor and introduced adaptive control over PN junction properties with only an applied lateral electric field bias. We anticipate an adaptive PN junction capability can be adopted into diodes. Furthermore, the manipulation of the physical characteristics of Galinstan is a precursor to flexible devices. Liquid-metal Galinstan can be embedded in microfluidic enclosures and exhibit shape deformability. There are many devices that can result from reconfigurability such as wearable diagnostics and conformal RF devices. Moreover, the liquid state of honey provides the potential for uniform and flexible gate dielectrics that are currently an issue for PVD-based gate dielectrics. In this paper, the authors only demonstrated a rigid architecture with inexpensive materials. The authors encourage the readers to explore alternate embodiments utilizing the liquid materials described and further explore the potential for flexible applications. The authors admit the use of liquid-metal Galinstan and honey for graphene devices in this paper was discovered by accident. We predict our transistors will lead towards the exploration of alternative materials that are slightly unconventional in the hopes these innovative discoveries provide a new class of materials that are non-toxic, biodegradable, and require minimal preparation time.

## Methods

### Liquid-metal Graphene Field-effect Transistor Fabrication

In this work, graphene was commercially acquired and a quality measure was conducted with a Renishaw InVia 514.5 *nm* (Green) Micro-Raman Spectroscopy System for three different graphene sites. The absence of a defect band *D* and analysis of the peak intensity ratio of the 2*D* and *G* Bands (*I*_2*D*_/*I*_*G*_ ≈ 2) indicated high quality graphene in all three sites, Fig. 1b. With high-quality monolayer graphene identified, a strip of graphene on Polyethylene Terephthalate (PET) was cut with standard cutting tools and adhered onto a glass microscope slide with the graphene side upward and PET side downward. Liquid-metal Galinstan droplets with volume 0.6 *mm* ^3^ each were then dispensed with a blunt-tip syringe to act as source and drain electrodes. Honey was commercially acquired and dispensed from a plastic dropper at a volume of 1.0 *mm* ^3^ between the two liquid-metal droplets to act as the electrolytic gate dielectric. For the PN junction *LM-GFET* embodiment, two wires were suspended above graphene and inside the honey gate dielectric. Both wires were biased with an Agilent E3648A Dual Power Supply. Current, voltage, and capacitance measurements were performed with an Agilent 4155C Semiconductor Parameter Analyzer, probe station, and Hioki IM3570 Impedance Analyzer in air and in a standard laboratory environment.

### Ion Gel Synthesis

Due to the toxicity and degradation of the ion gel components in contact with atmospheric oxygen, synthesis was conducted in a nitrogen-purged atmospheric controlled glove box (Vacuum Atmospheres Company OMNI-LAB). Only when the glove box oxygen content was reduced to below 2 ppm, 1-Ethyl-3 Methylimidizalium Bis(Trifluoromethylsufonyl)imide ([EMIM][TFSI]) ionic liquid (weight 0.467 g, measured with a Fisher Scientific Microbalance) was mixed with Polystyrene-Poly (ethylene oxide) (PS-PEO) diblock copolymer (weight 0.036 g) using a magnetic stirrer. 5.8 mL of acetonitrile solvent (weight 4.56 g) was added with a standard syringe needle to the ionic liquid/copolymer solution to thoroughly dissolve the copolymer. The final ion gel solution consisted of 9.21% [EMIM][TFSI] ionic liquid, 0.71% PS-PEO copolymer, and 90.07% acetonitrile solvent that was evaporated before I-V measurements. The ion gel was dispensed on the *LM-GFET* devices with a plastic dropper and measurements were all conducted in a fume hood to reduce exposure to the ion gel.
